# Comparative Evaluation of Modified Wasserstein GAN-GP and State-of-the-Art GAN Models for Synthesizing Agricultural Weed Images in RGB and Infrared Domain

**DOI:** 10.1016/j.mex.2025.103309

**Published:** 2025-04-09

**Authors:** Shubham Rana, Matteo Gatti

**Affiliations:** Department of Sustainable Crop Production, Università Cattolica del Sacro Cuore, Via Emilia Parmense 84, Piacenza, 29122, Italy

**Keywords:** Generative adversarial network (GAN), Wasserstein generative adversarial network (WGAN), Gradient Penalty (GP), *Raphanus raphanistrum*, Modified Wasserstein Generative Adversarial Network with Gradient Penalty

## Abstract

This study investigates the application of modified Wasserstein Generative Adversarial Networks with Gradient Penalty (WGAN-GP) to generate synthetic RGB and infrared (IR) datasets to meet the annotation requirements for wild radish (Raphanus raphanistrum). The RafanoSet dataset was used for evaluation. Traditional WGAN models struggle with vanishing gradients and poor convergence, affecting data quality. Customizations in WGAN-GP improved synthetic image quality, especially in maintaining SSIM for RGB datasets. However, generating high-quality IR images remains challenging due to spectral complexities, with lower SSIM scores. Architectural enhancements including transposed convolutions, dropout, and selective batch normalization improved SSIM scores from 0.5364 to 0.6615 for RGB and from 0.3306 to 0.4154 for IR images. This study highlights the customized model's key features:

• Produces a 128 × 7 × 7 tensor, optimizes feature map size for subsequent layers, with two layers using 4 × 4 kernels and 128 and 64 filters for upsampling.
• Uses 3 × 3 kernels in all convolutional layers to capture fine-grained spatial features, incorporates batch normalization for training stability, and applies dropout to reduce overfitting and improve generalization.

## Graphical abstract

**Figure:**
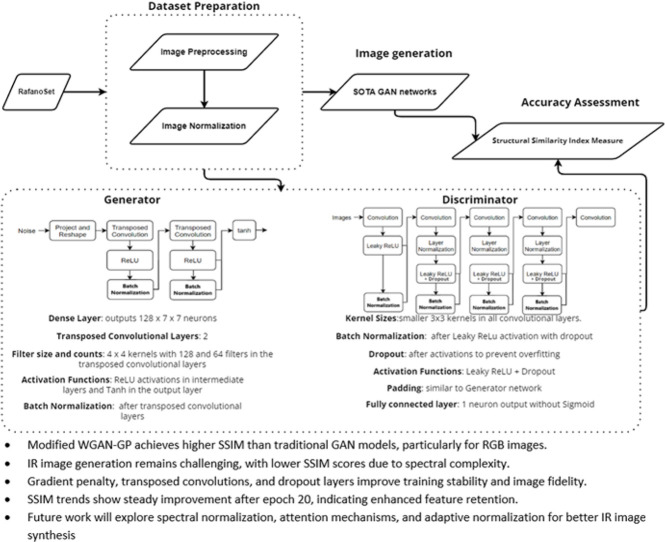

Specifications table

**Table:** 

Subject area:	Computer Science
More specific subject area:	Digital Image Processing
Name of your method:	Modified Wasserstein Generative Adversarial Network with Gradient Penalty
Name and reference of original method:	I. Gulrajani, F. Ahmed, M. Arjovsky, V. Dumoulin, and A. Courville, Improved training of Wasserstein GANs, arXiv:1704.00028v3.(2017). https://doi.org/10.48550/arXiv.1704.00028.
Resource availability:	https://doi.org/10.5281/zenodo.10567783

## Background

The proposed methodology in this study is based on the customization, application, and comparative analysis of a Modified Wasserstein Generative Adversarial Network with Gradient Penalty (WGAN-GP) against other state-of-the-art GAN models to meet data augmentation requirements [[1], [2], [3]]. The experiment is focused on generating proximally acquired RGB and Infrared (IR) images for *Raphanus raphanistrum* weed in RGB and IR spectrum and provide a qualitative and quantitative assessment in terms of Structural Similarity Index Measure (SSIM) with other GAN models.

**Fig. 1:**
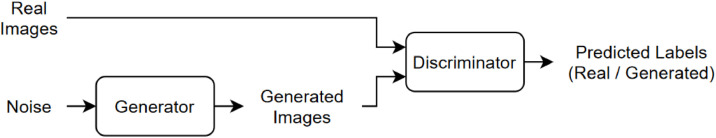
Basic Structure of a GAN.

In precision agriculture, computer vision-based weed management remains a challenging task due to the heterogeneity created by weed infestation among crops and the associated spectral overlap, which makes the localization of weeds difficult, particularly for weeds like *Raphanus raphanistrum* [4,5]. Accurate identification is highly dependent on morphological similarities between crops and weeds, particularly in situations of spectral overlap, variability in environmental conditions, and limitations in spectral data quality [4]. These factors necessitate advanced tools capable of enhancing dataset quality for training robust machine learning models. GANs consist of two neural networks - the generator and the discriminator, working simultaneously (Fig. 1).

Traditional GANs have shown potential in data augmentation but often encounter issues like vanishing gradients, mode collapse, and poor convergence, particularly when applied to complex datasets [6,7]. The introduction of WGAN-GP, which leverages the Wasserstein distance and incorporates a gradient penalty, represents a significant advancement in overcoming these challenges, ensuring more stable training and higher-quality synthetic data [2,7].

The architectural modifications proposed in this study, including the integration of convolutional layers, transposed convolutions, and batch normalization are tailored to enhance the fidelity and structural integrity of generated images across both spectral domains. These enhancements address the limitations of fully connected layers, enabling the preservation of spatial relationships critical for synthetic image generation [7]. The inclusion of transposed convolutions ensures artifact-free upsampling, which is a prerequisite to maintain image coherence in RGB and IR datasets [8].

The methodology also introduces batch normalization to stabilize training dynamics and improve convergence, while deliberately omitting it in the discriminator to maximize the effectiveness of the gradient penalty [9]. These design choices collectively contribute to generating high-quality synthetic datasets, as evidenced by improved Structural Similarity Index (SSIM) scores compared to traditional GAN models [4]. The utility of the modified WGAN-GP methodology can be extended beyond generating synthetic images for weed detection. By generating synthetic images across different growth stages, lighting conditions, and morphological variations, this methodology enhances model generalizability and robustness [10].

Moreover, the ability of this methodology to generate high-quality annotated synthetic data facilitates advancements in segmentation tasks, domain adaptation, and class balancing. These capabilities are instrumental in improving the performance of machine learning models across diverse agricultural applications, including crop monitoring and management [11]. While the study demonstrates the effectiveness of the modified WGAN-GP in generating RGB datasets, the relatively lower performance on IR datasets highlights the need for further refinements. Future work could explore integrating attention mechanisms, advanced loss functions, and spectral normalization techniques to enhance spectral data synthesis [12,13].

Despite so much progress in GAN-based data augmentation in precision agriculture, there are limitations which remain unaddressed in current literature. In an earlier work, [14] proposed an object-centric GAN strategy that inserted synthetic crop patches into real images to augment training data for semantic segmentation. While the approach effectively reduced annotation costs and improved segmentation accuracy, it was limited by its focus solely on crop generation, excluding weeds—despite their critical role in segmentation tasks. This contributed to consistently low weed intersection over union scores (below 0.40), indicating inadequate learning of minority class features. Additionally, the technique sometimes introduced domain shifts due to style mismatches between synthetic objects and real backgrounds. Another limitation was the absence of multispectral or infrared image generation, which is increasingly relevant for advanced sensing in agriculture.

Another work, [15] introduced a shape-and-style-based GAN augmentation framework for multispectral data, achieving notable mIoU improvements. However, their model frequently reused the same plant shapes during training which reduces the diversity and limits the generalizability of synthetic samples. Additionally, their augmentation was restricted to only central part of the plant patches and neglects edge cases or complex inter-plant interactions in cluttered scenes. While their model successfully controlled shape and texture, it lacked spatial semantic consistency with the surrounding soil and illumination conditions, raising concerns about scene-level coherence. Furthermore, the model was tested exclusively on sugar beet crops, leaving its scalability to other crop species uncertain.

Similarly, [16] used a conditional GAN - Pix2Pix for generating synthetic fakes of maize and common weeds but faced challenges in replicating fine-grained textural features, particularly for visually complex species like Shepherd's Purse. While shape information was preserved, stylistic artifacts and inconsistencies in background textures compromised the structural similarity of the generated images. Additionally, the model produced outputs at a relatively low resolution of 256 × 256, which may not suffice for high-resolution field robotics or real-time agricultural applications. The study also did not address variability due to lighting conditions or crop growth stages, which are essential for robust deployment in real-world scenarios.

Unlike previous GAN-based approaches for agricultural image synthesis, this study has introduced modified WGAN-GP architecture optimized for both RGB and IR weed images, demonstrating improved structural fidelity across modalities and has taken diverse scales and illumination conditions. The experiment has also considered heterogeneity in terms of class shape, posture, occlusion and spectra for demonstrating the performance of the customized architecture. Collectively, we have handled spectral variation, ensured contextual coherence in different images, and enhanced the representation of weeds in all modalities.

## Method details

### Dataset preparation

**Fig. 2:**
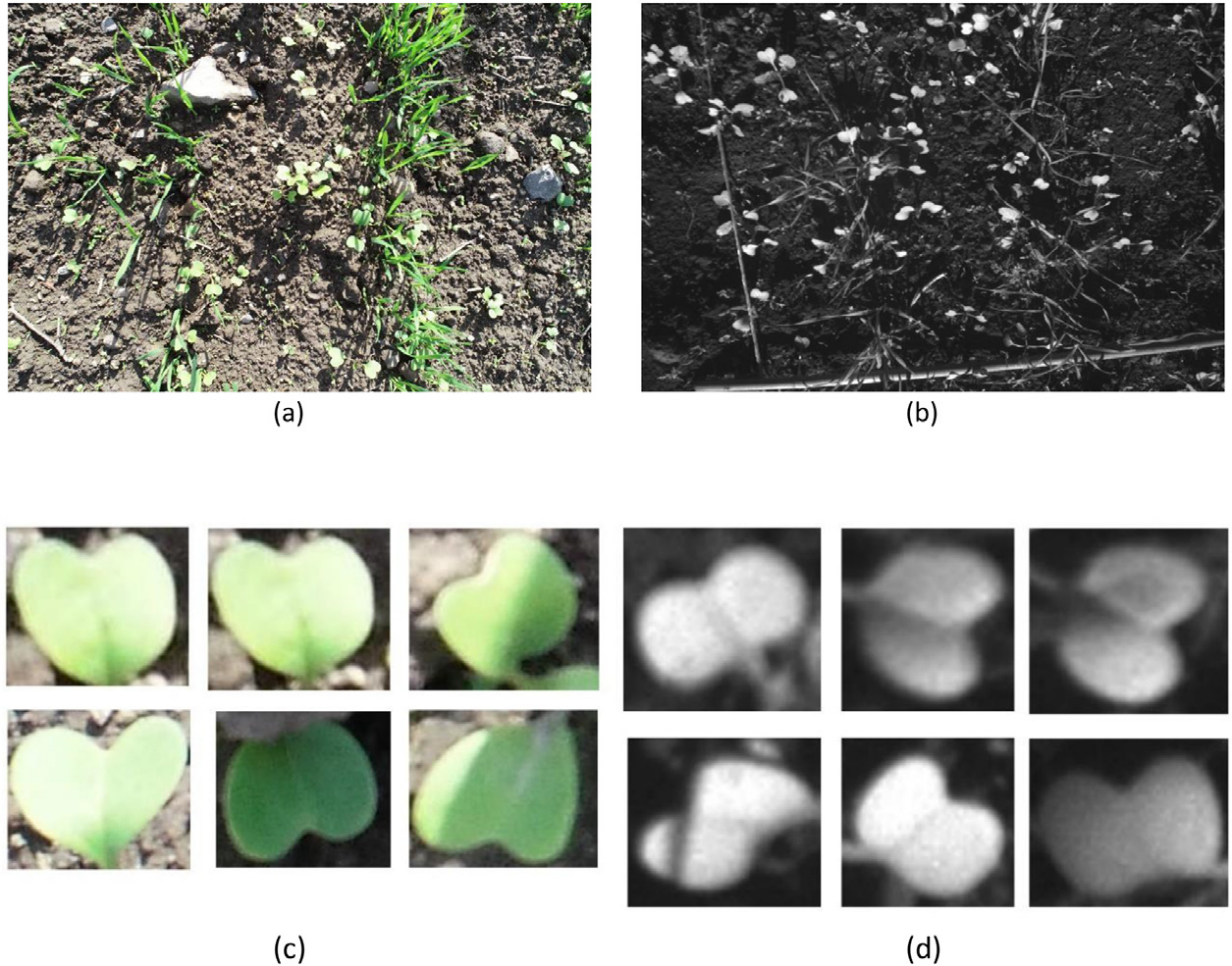
(L-R & Top-Bottom).

Multiple leaf-bound instances were clipped from the 12 January 2022 RGB and 13 January 2022 Red Edge bands [17] to constitute two classes of data towards training the Wasserstein GAN with Gradient Penalty [17]. A few clipped samples from the datasets are demonstrated in Fig. 2(c) and (d).

(a) RGB image instance (1 m) containing a heterogeneous mix of *Triticum aestivum* (wheat) and *Raphanus raphanistrum* (horseradish)
(b) Infrared instance (1 m) containing a heterogeneous mix of *Triticum aestivum* (wheat) and *Raphanus raphanistrum* (horseradish)
(c) Clipped samples from (a) at 155 × 115 pixels
(d) Clipped samples from (b) at 155 × 115 pixels

### Image collection and preprocessing

A total of 57 RGB images were acquired on 12 January 2022. 876 leaf-bound samples were clipped in the order of 155 × 115 pixels containing the periphery of *Raphanus raphanistrum* [17]. The acquisition sensor was Canon 2000D and the sensing altitude was 1 m. A total of 202 Infrared images were acquired on 13 January 2022. 850 leaf-bound samples were clipped in the order of 155 × 115 pixels containing the periphery of *Raphanus raphanistrum*. The acquisition sensor was Micasense RedEdge–M and the sensing altitude was 1 m. Both the datasets were split into training (80 %) and testing (20 %).

### Image normalization

Image normalization is a critical preprocessing step for training neural networks, including generator networks, and it involves scaling pixel values to a common range to improve convergence and stability. For RGB images, normalization typically involves rescaling pixel values from their original range (e.g., 0–255) to a range of 0–1 or −1 to 1. This can be done by dividing the pixel values by 255 for a 0–1 range, or by first rescaling to 0–1 and then shifting and scaling to achieve a −1 to 1 range. Additionally, channel-wise mean subtraction is commonly performed, where the mean and standard deviation for each channel (R, G, B) are computed from the training dataset. Each pixel value in a channel is then subtracted by the channel's meaning and divided by the channel's standard deviation [18,19].

For Multispectral (MS) images, the normalization techniques are similar but must account for the additional spectral bands beyond the standard RGB channels. MS images' pixel values are also rescaled to a range of 0–1. Band-wise normalization involves calculating the mean and standard deviation for each spectral band across the entire training dataset and then normalizing each band separately by subtracting its meaning and dividing by its standard deviation. Min-max normalization is another common approach for MS images, particularly useful for bands with varying value ranges, where each band is scaled to the 0–1 range based on its minimum and maximum values [[20], [21], [22]].

### Rationale for network modifications

**Gradient Penalty**: The inclusion of a gradient penalty enforces the Lipschitz constraint on the discriminator, stabilizing training by smoothing the optimization landscape. This eliminates the need for weight clipping, which can overly restrict the model's capacity [2]. The gradient penalty requires retainment in the discriminator to enforce the Lipschitz constraint, which is crucial for stabilizing the adversarial training process [23,24]. In the modified architecture, the penalty will be applied in conjunction with convolutional layers to enhance its effectiveness for spatially structured data such as RGB and IR images.

**Convolutional Layers**: Replacing fully connected layers with convolutional layers preserves spatial relationships in the image data, a critical factor in generating high-quality synthetic images. This design enhances the model's ability to capture and replicate fine structural details [6]. In the generator, fully connected layers will be replaced with convolutional and transposed convolutional layers to enhance spatial feature preservation. These transposed convolutions need to be configured with a filter size of 4 × 4, a stride of 2, and same padding to ensure artifact-free upsampling. Batch normalization will be applied to stabilize training and improve convergence. In the discriminator, multi-layer convolutional architecture with Leaky ReLU activation and dropout needs to be implemented to improve feature extraction and prevent overfitting.

**Transposed Convolutions**: Transposed convolutions ensure that generated images maintain a coherent structure, avoiding artifacts commonly seen with simpler upsampling techniques [25]. The generator network requires transposed convolutions to progressively upsample feature maps from a low-resolution latent space to the final image resolution. Each transposed convolution layer needs to employ 4 × 4 filters, a stride of 2, and the same padding to ensure spatial consistency and reduce artifacts. Batch normalization and ReLU activation would be applied after each layer to stabilize training and enhance feature generation. The final layer to be used is Tanh activation to normalize pixel values to the range [−1, 1].

**Batch Normalization**: Batch normalization stabilizes training by normalizing feature distributions, reducing internal covariate shifts, and improving convergence [26]. The integration of this function in the generator will improve the training stability, which will be evidenced by smoother convergence in the loss curves. Generated images would be demonstrating higher structural fidelity and reduced artifacts, particularly in RGB datasets. The decision to omit batch normalization in the discriminator will ensure the gradient penalty's effectiveness, preserving the model's stability.

## Model design

**Generator Network:** The generator network in the updated Wasserstein GAN with Gradient Penalty (WGAN-GP) is designed to produce RGB images from random noise (Table 1). It begins with a fully connected layer that transforms the 100-dimensional latent vector into a 128 × 7 × 7 feature map using the ReLU activation function for non-linearity. This feature map is then reshaped into a 7 × 7 × 128 format and undergoes a series of upsampling operations via transposed convolution layers. The first transposed convolution layer applies 128 filters of size 4 × 4 with a stride of 2 and same padding, doubling the spatial dimensions to 14 × 14 while maintaining a depth of 128. Batch normalization is used to stabilize and speed up training, followed by ReLU activation. The second transposed convolution layer further upsamples the feature map to 28 × 28 with 64 filters of size 4 × 4, again followed by batch normalization and ReLU activation. The final output layer is a convolutional layer with 3 filters (for RGB) of size 4 × 4 and same padding, using the Tanh activation function to scale pixel values to the range [−1, 1]. This configuration enables the generator to produce high-quality RGB images that closely resemble the real images in the training dataset (Table 2).

**Table 1:** Modified Architecture of WGAN–GP Generator Network.

Layer	Type	Parameters	Activation Function
1	Fully Connected	128 × 7 × 7 neurons	ReLU
2	Reshape	7 × 7 × 128	**-**
3	Transposed Convolution	4 × 4, 128 filters, stride 2, same padding	BatchNorm + ReLU
4	Transposed Convolution	4 × 4, 64 filters, stride 2, same padding	BatchNorm + ReLU
5	Convolution	4 × 4, 3 filters, same padding	Tanh

**Table 2:** Modified Architecture of WGAN–GP Discriminator Network.

Layer	Type	Parameters	Activation Function
1	Convolution	3 × 3, 16 filters, stride 2, same padding	Leaky ReLU + Dropout
2	Convolution	3 × 3, 32 filters, stride 2, same padding	BatchNorm + Leaky ReLU + Dropout
3	Convolution	3 × 3, 64 filters, stride 2, same padding	BatchNorm + Leaky ReLU + Dropout
	Convolution	3 × 3, 128 filters, stride 1, same padding	BatchNorm + Leaky ReLU + Dropout
5	Fully Connected	1 neuron	**–**

**Discriminator Network:** The discriminator network in this WGAN–GP is designed to differentiate beween real RGB images and those generated by the generator. It begins with a convolutional layer that applies 16 filters of size 3 × 3 with a stride of 2 and same padding, reducing the spatial dimensions while preserving the depth. This layer uses the Leaky ReLU activation function to handle negative values and a dropout layer to prevent overfitting. The subsequent convolutional layer applies 32 filters of size 3 × 3 with a stride of 2 and same padding, followed by batch normalization, Leaky ReLU activation, and another dropout layer. The third convolutional layer further reduces the spatial dimensions using 64 filters of size 3 × 3 with a stride of 2 and same padding, again followed by batch normalization, Leaky ReLU activation, and dropout. The final convolutional layer applies 128 filters of size 3 × 3 with a stride of 1 and same padding, followed by batch normalization and Leaky ReLU activation. This results in a deep feature map which is then flattened and passed through a fully connected layer to produce a single output value. This architecture enables the discriminator to effectively learn the features that distinguish real images from fake ones generated by the generator, providing accurate feedback to improve the generator's performance during training.

### Training the WGAN

The training begins with the careful setting of hyperparameters. The latent dimension, which represents the input noise vector's size for the generator, is typically set to 100, allowing for a sufficient level of complexity in the generated images. A batch size of 32 is selected to balance memory usage and training stability, and the training process is set to continue for a maximum of 50 epochs, ensuring ample opportunity for the network to learn. The learning rate is fixed at 0.00005 to ensure gradual and stable updates to the network parameters, while the discriminator's weights are clipped to the range of [−0.01, 0.01] to enforce the Lipschitz constraint essential for WGAN stability [23,24].

During training, the discriminator is updated more frequently than the generator, typically five times per generator update. This approach ensures the discriminator maintains its ability to effectively distinguish between real and synthetic images, providing a robust gradient signal for the generator's updates. The optimization process for both networks employs the RMSProp algorithm to handle the non-stationary settings and smoothen the weight updates required by WGANs [27]. The training process is monitored by observing the loss functions of both the generator and the discriminator to ensure stability and convergence. The generator aims to minimize the negative log- likelihood of the discriminator's classification, while the discriminator's objective is to maximize its ability to differentiate between real and synthetic im-ages. This careful balance of hyperparameters and training dynamics is required to train a WGAN capable of producing high-quality synthetic RGB and IR images of agricultural weeds.

### Qualitative and quantitative assessment

The Structural Similarity Index (SSIM) is a technique for evaluating the similarity between two images. It is utilized to determine the quality of an image in comparison to a reference image. Unlike traditional metrics such as Mean Squared Error (MSE) or Peak Signal-to-Noise Ratio (PSNR), SSIM considers variations in structural infor-mation, luminance, and contrast, making it more consistent with human visual perception [[28], [29], [30]]. SSIM (Eq. (1)) is computed on different windows of an image. The similarity measure between two windows x and y of the same size is explained below:

(1) $$\mathrm{SSIM}\;\left(x,y\right)=\frac{\left(2\mu_{x}\mu_{y}+\;C_{1}\right)\left(2\sigma_{\mathrm{xy}}+\;C_{2}\right)}{\left(\mu_{x}^{2}+\mu_{y}^{2}+C_{1}\right)\left(\sigma_{x}^{2}+\sigma_{y}^{2}+C_{2}\right)}$$

Where, x and y are the two images being compared.

$\mu_{x}$ is the mean of image x; $\mu_{y}$ is the mean of image y.

$\sigma_{x}^{2}$ and $\sigma_{y}^{2\;}$are the variance of images x and y respectively

$\sigma_{\mathrm{xy}}$ is the covariance of images x and y respectively

$C_{1}={\left(K_{1}L\right)}^{2}$ and $C_{2}={\left(K_{2}L\right)}^{2}$ two constants to stabilize the division with weak denominator, where L is the dynamic range of the pixel-values (typically 255 for 8-bit images), and $K_{1}$ and $K_{2}$ are small constants.

### Method validation

I. Generated RGB images with Modified WGAN-GP

#### SSIM between the training images and the generated images

The SSIM of 0.5364 between the training images and the generated image shows that the generated images have a lower similarity to the training images (Fig. 3(a)). This relatively low SSIM value suggests that the generated image does not closely replicate the features, textures, or overall structure of the training image, indicating that the generation process might have introduced significant deviations from the original data (Fig. 3(b)).

**Fig. 3(a):**
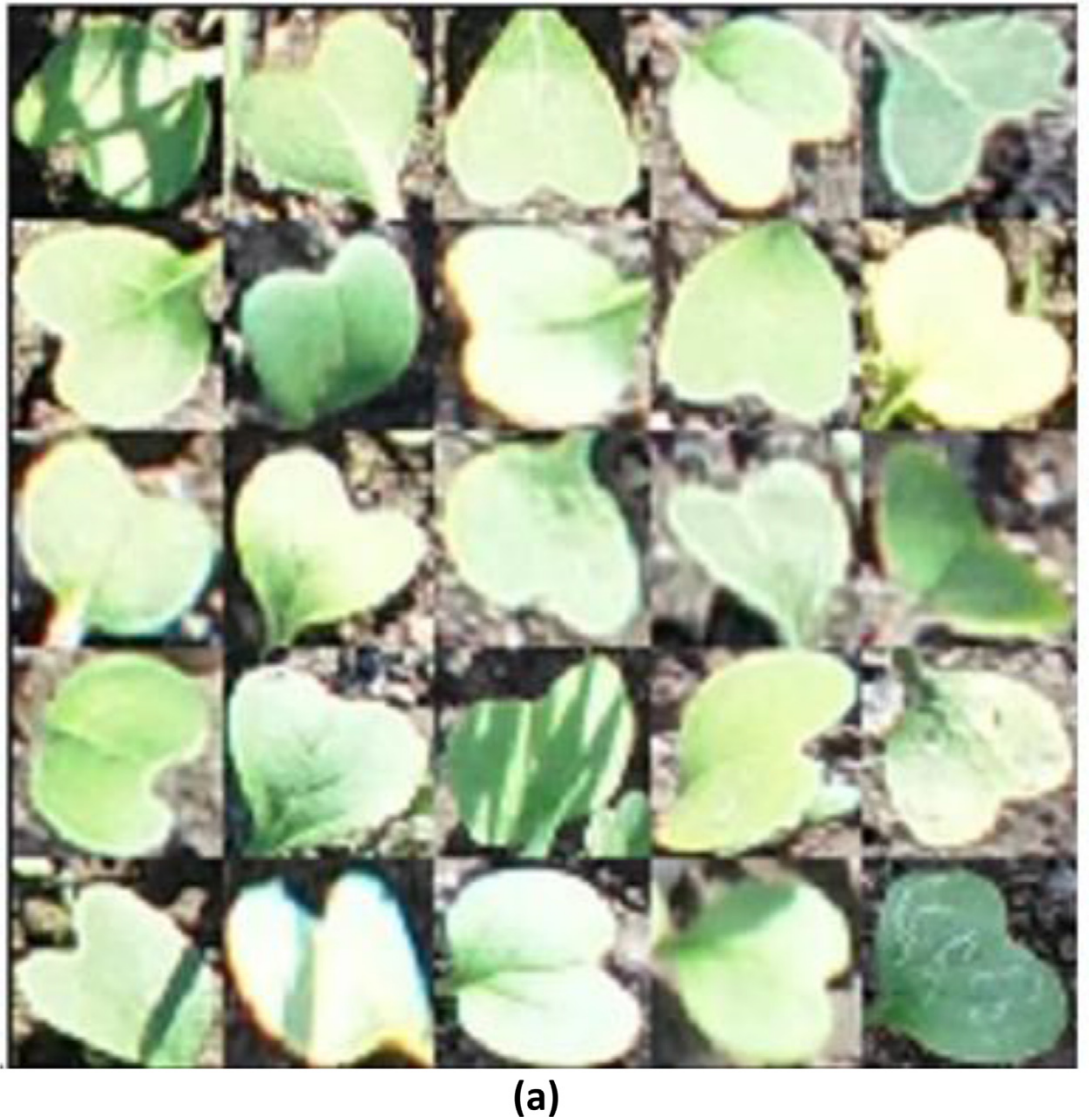
Training Images.

**Fig. 3(b):**
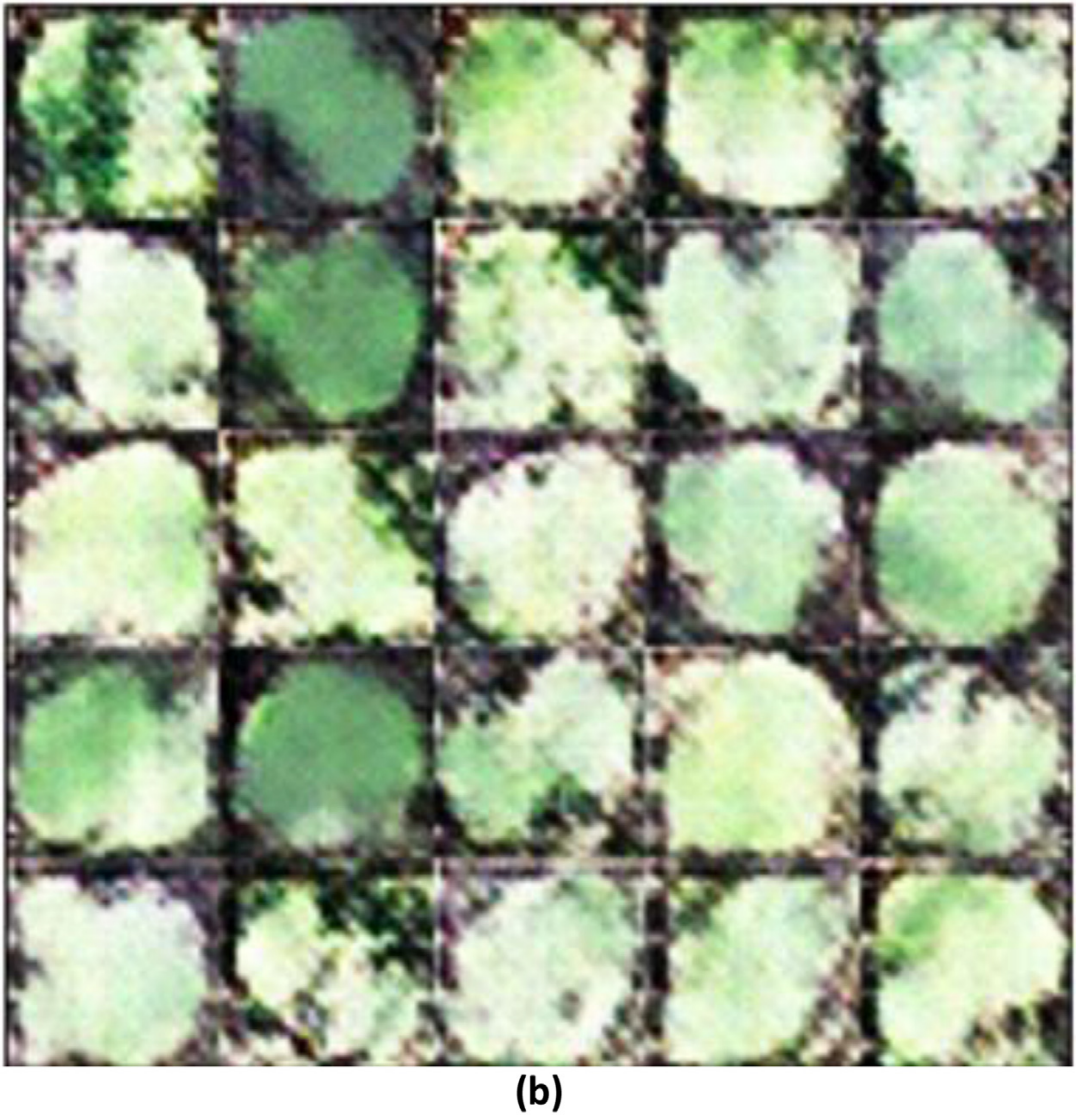
Generated Images using WGAN-GP.

While the model was somewhat successful in capturing the general structure and appearance of the Raphanus leaves, it struggled with finer details and maintaining consistent texture and luminance across the images. The gradient penalty helps in stabilizing the training of the WGAN, but it may not always lead to perfect reconstructions, especially in cases where the dataset has high variability or when the model needs further tuning.

#### SSIM between the training images and the generated images with modified wgan-gp

The image generated using a modified WGAN-GP, exhibits a noticeable improvement. The leaves in this image have clearer boundaries and somewhat better-defined shapes compared to the image generated, which is reflected in the higher SSIM of 0.6615 (Fig. 3(c)). However, the image still suffers from some noise and loss of fine detail, indicating that while the modifications to the WGAN were beneficial, there is still room for further enhancement. This suggests that the modified architecture of WGAN-GP was more successful in capturing the features of the training image compared to the initial generation process.

II. **Generated Infrared Images with Modified WGAN**–**GP**

**Fig. 3(c):**
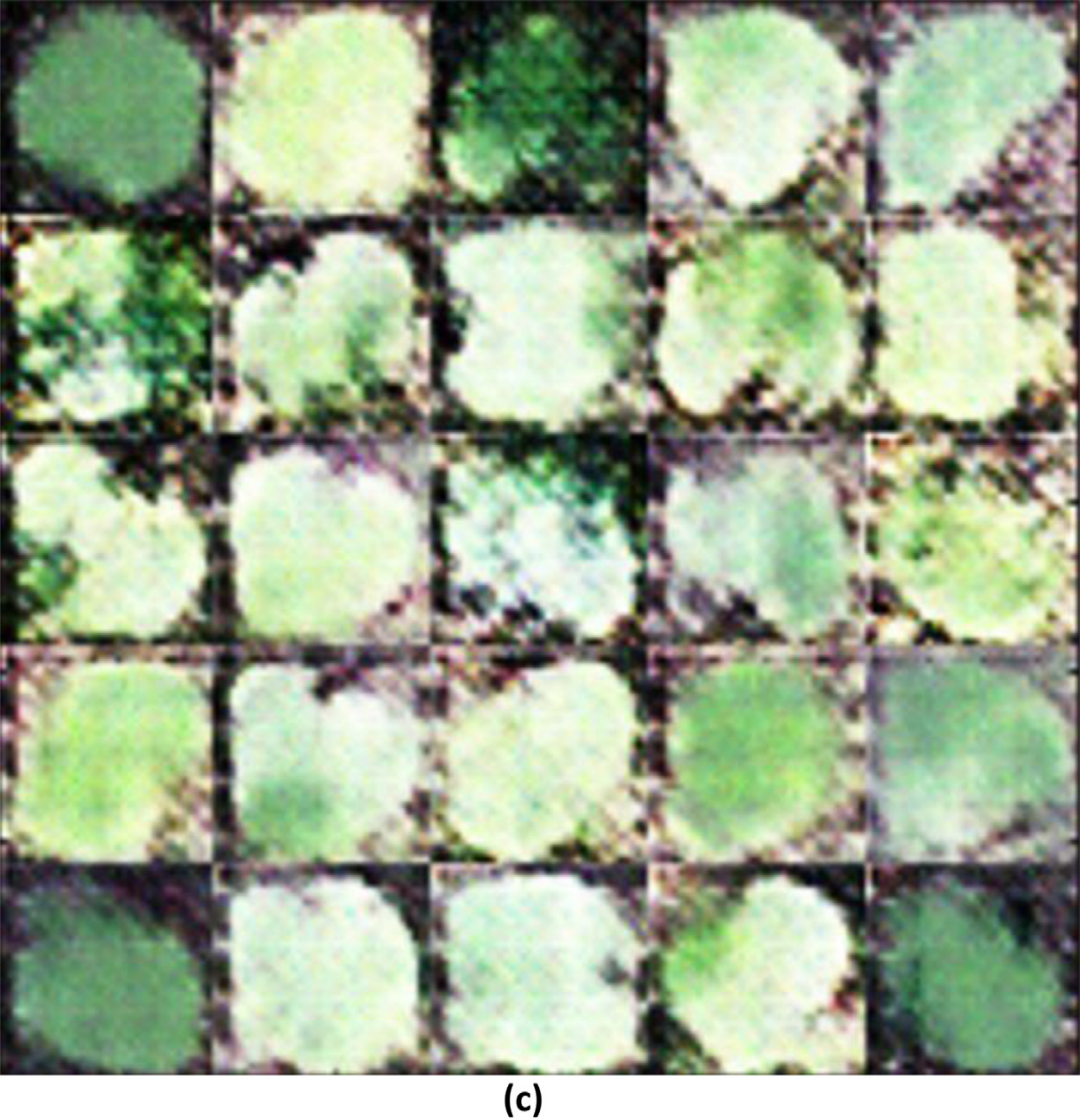
Generated images using modified WGAN-GP.

### SSIM between the training images and the generated images

The SSIM of 0.3306 between the generated image and the original training image shows a slightly lower level of similarity (Fig. 4(a), (b)). This indicates that while the generated image attempts to replicate the training image, there are noticeable differences in terms of structure, contrast, and luminance. This lower SSIM value compared to the reconstructed image's SSIM with the training image suggests that the generated image is less accurate in representing the features of the original dataset.

**Fig. 4(a):**
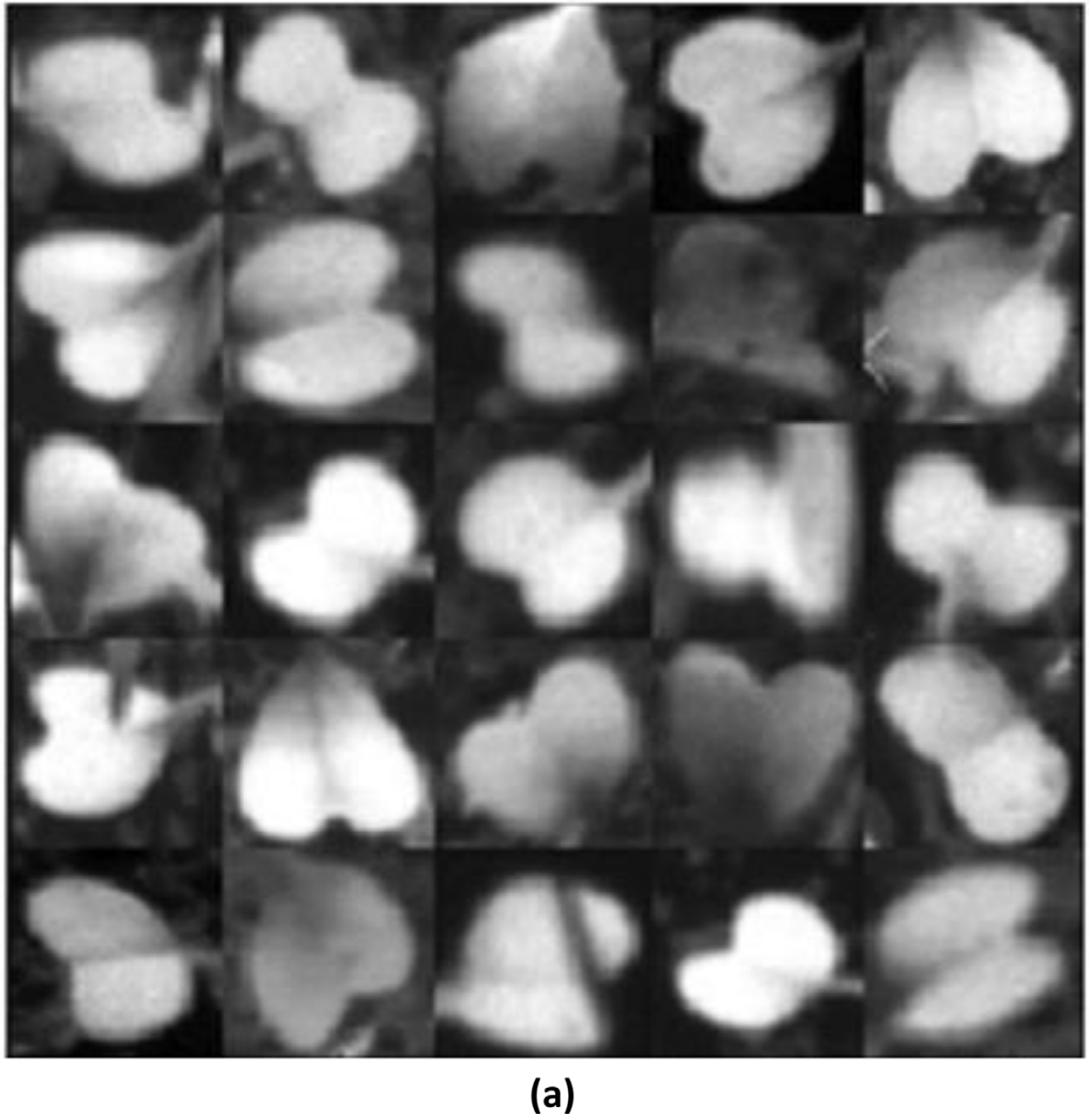
Training Images.

**Fig. 4(b):**
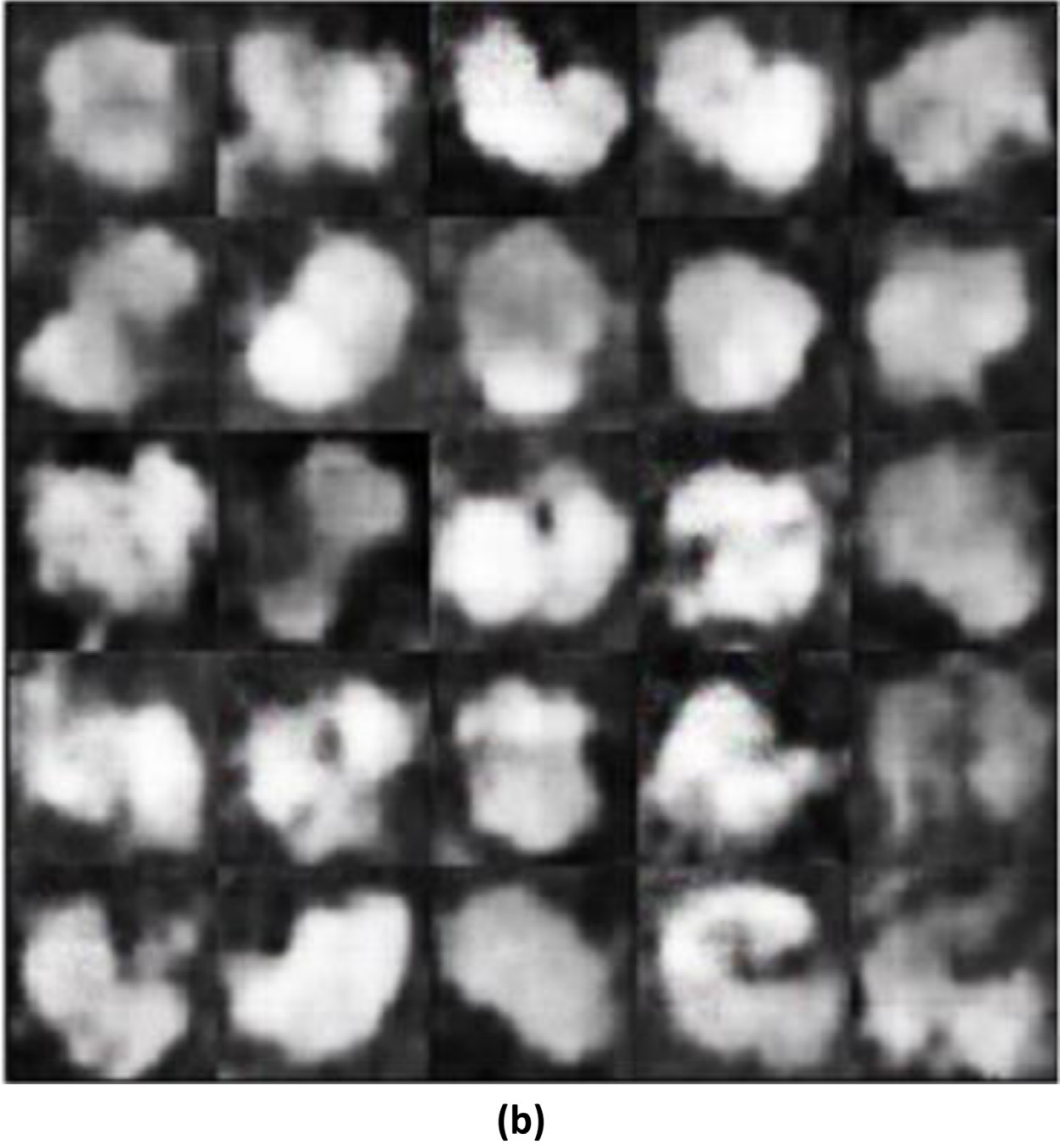
Generated Images using WGAN-GP.

### SSIM between the reconstructed image and the training image

The highest SSIM value of 0.4154 is observed between the generated images using a modified WGAN with gradient penalty and the training images (Fig. 4(c)). This improvement in SSIM from the generated image in step 4.2.1 (relative to the training images) indicates that the architectural modification has succeeded in enhancing the fidelity of the generated images with respect to the original training data.

III. **Comparative Analysis of Modified Wasserstein GAN-GP with other GAN architectures**

**Fig. 4(c):**
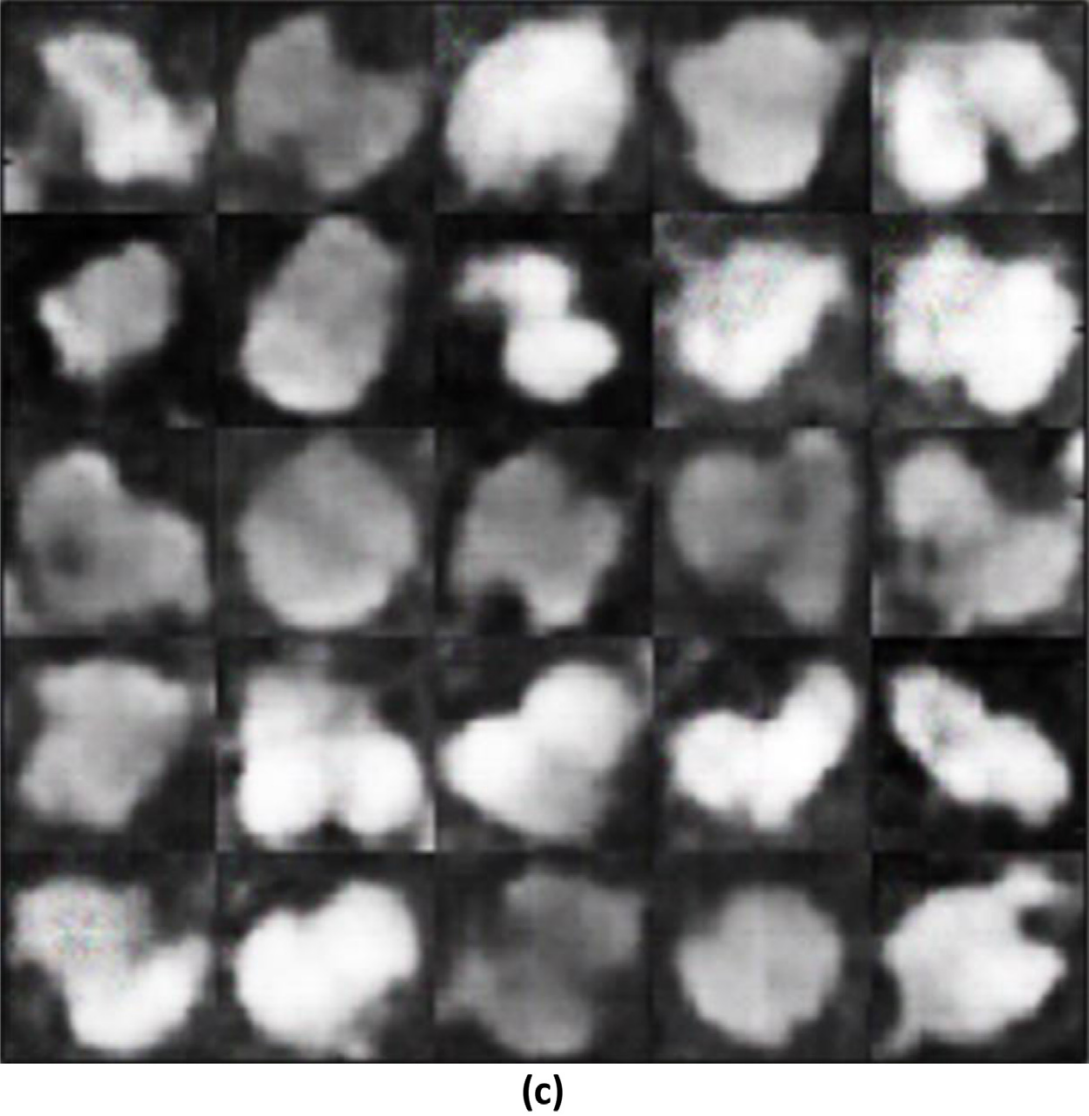
Generated images using modified WGAN-GP.

**Fig. 5:**
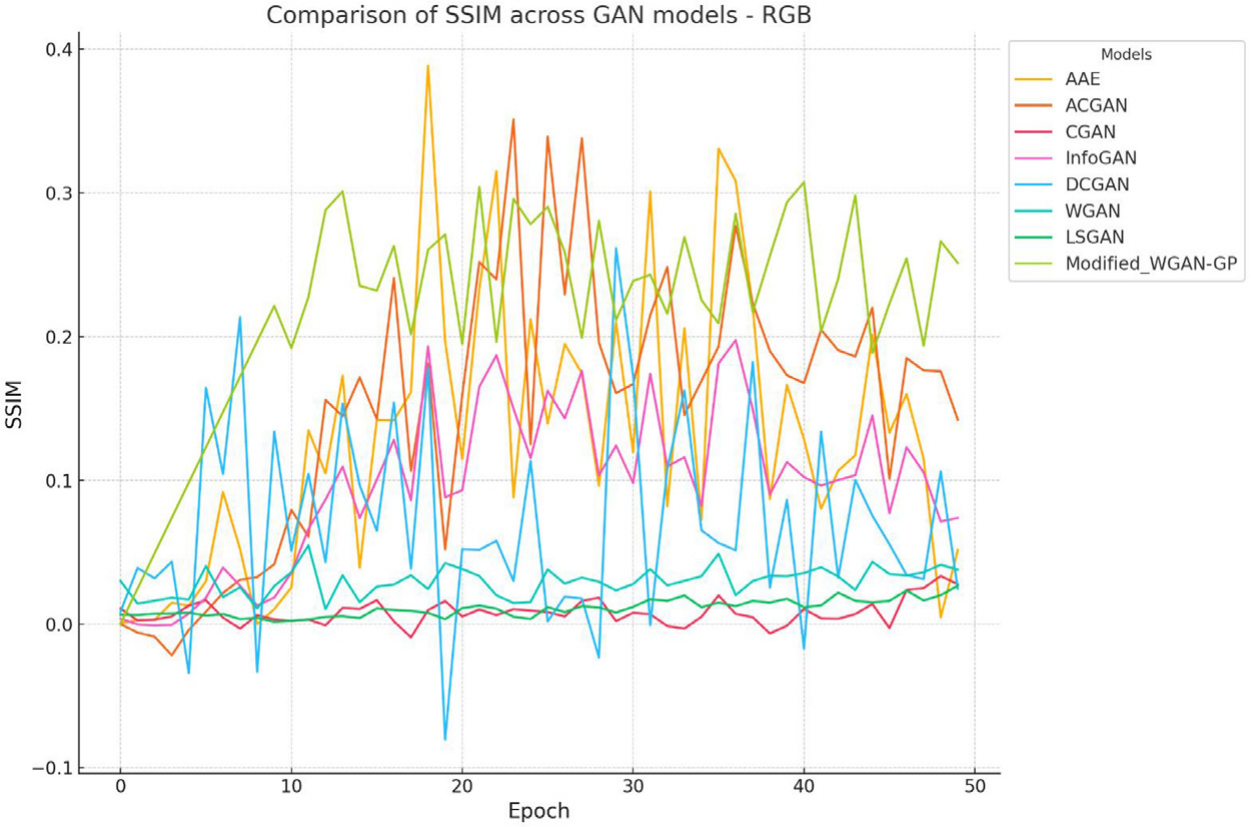
Comparative analysis of SSIM for different GAN architectures for generated RGB instances.

The SSIM (Structural Similarity Index Measure) values across 50 epochs for various GAN models are illustrated in Fig. 5. The Modified Wasserstein GAN with Gradient Penalty (Modified WGAN-GP) exhibits consistently higher SSIM values, particularly after epoch 20, signifying its superior capacity to optimize structural similarity. This steady improvement is attributed to architectural refinements, including gradient penalties and convolutional layers, which enhance training stability and enable the model to capture fine-grained details [1,31]. Despite its strengths, occasional peaks and dips in SSIM values reveal training variability, potentially caused by sensitivity to hyperparameters [24,31].

The Adversarial Autoencoder (AAE) demonstrates significant fluctuations during training, with sharp SSIM peaks in early epochs [32]. While these peaks highlight potential, instability emphasizes the need for better tuning of auxiliary learning tasks, such as adversarial autoencoding. This contrasts with the steadier progression of Modified WGAN-GP, which underscores its robust training dynamics.

Traditional GAN models, including CGAN [33], InfoGAN [34], DCGAN [6], WGAN [1], and LSGAN [35], consistently underperform, showing flat trends and low SSIM values. Their lack of advanced architectural features, such as gradient penalties or attention mechanisms, limits their ability to capture and preserve structural details. These findings emphasize the need for further innovation to enhance their performance.

**Fig. 6:**
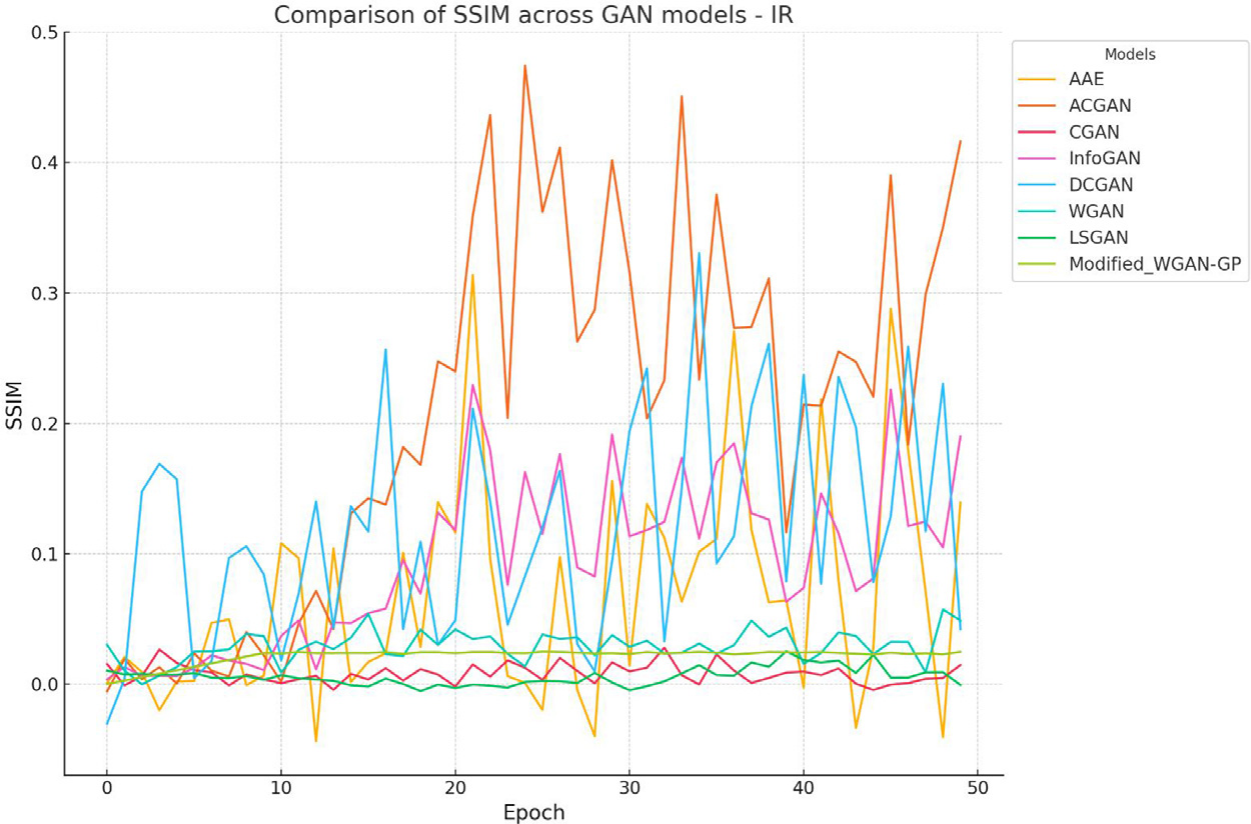
Comparative analysis of SSIM for different GAN architectures for generated IR instances.

Fig. 6 highlights SSIM values across 50 epochs for various GAN models applied to IR datasets. Generating high-quality synthetic IR images remains challenging, as demonstrated by the lower SSIM values across all models compared to RGB datasets. The Modified WGAN-GP delivers the highest SSIM values, particularly after epoch 20, showcasing its relative effectiveness in maintaining structural similarity despite the inherent spectral complexities of IR data [12,3]. However, its performance reveals more variability than on RGB datasets, indicating a need for further architectural refinement to handle spectral noise and reduced contrast in IR images [20,36].

AAE and ACGAN exhibit occasional SSIM spikes during training, reflecting their potential but also their instability when applied to IR datasets [26,34]. These fluctuations highlight challenges in achieving consistent performance for IR images, possibly due to differences in data characteristics, such as spectral noise or structural complexity [4].

Traditional GAN models—CGAN [33], InfoGAN [34], DCGAN [6], WGAN [1], and LSGAN [35]—maintain consistently low SSIM values for IR datasets, with minimal improvement across epochs. This reinforces their limited capacity to adapt to the spectral demands of IR data. The Modified WGAN-GP's relative stability and higher SSIM values underscore its advanced capabilities, though further refinements are needed to enhance its generalizability.

Across both RGB and IR datasets, the Modified WGAN-GP outperforms traditional models by generating images with higher SSIM values and improved structural integrity. However, IR data poses additional challenges, resulting in greater variability in SSIM trends [21]. While the Modified WGAN-GP exhibits stable improvements, occasional variability highlights the need for enhancements like spectral normalization or advanced loss balancing [24,31]. The consistent underperformance of models such as CGAN, InfoGAN, and LSGAN further underscores the importance of integrating advanced features, such as attention mechanisms, to improve their capabilities [37].

Overall, the Modified WGAN-GP demonstrates significant strengths in generating high-quality synthetic images for both RGB and IR datasets, with notable areas for further refinement. Its architectural enhancements open a room for future work, which should focus on addressing training variability and improving spectral consistency, especially for IR data. These advancements are vital for advancing synthetic data generation in precision agriculture.

### Observations

**Fig. 7:**
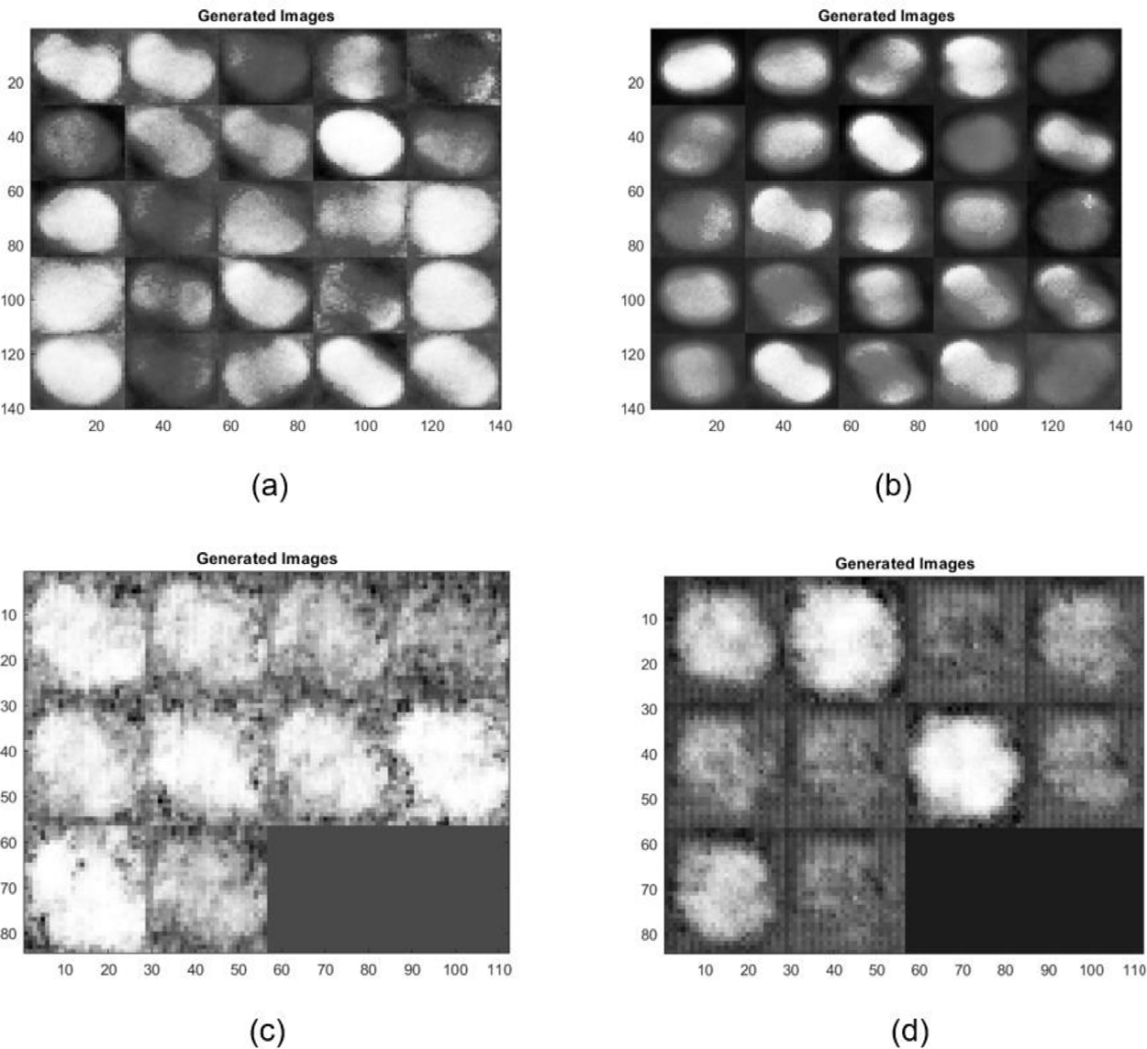

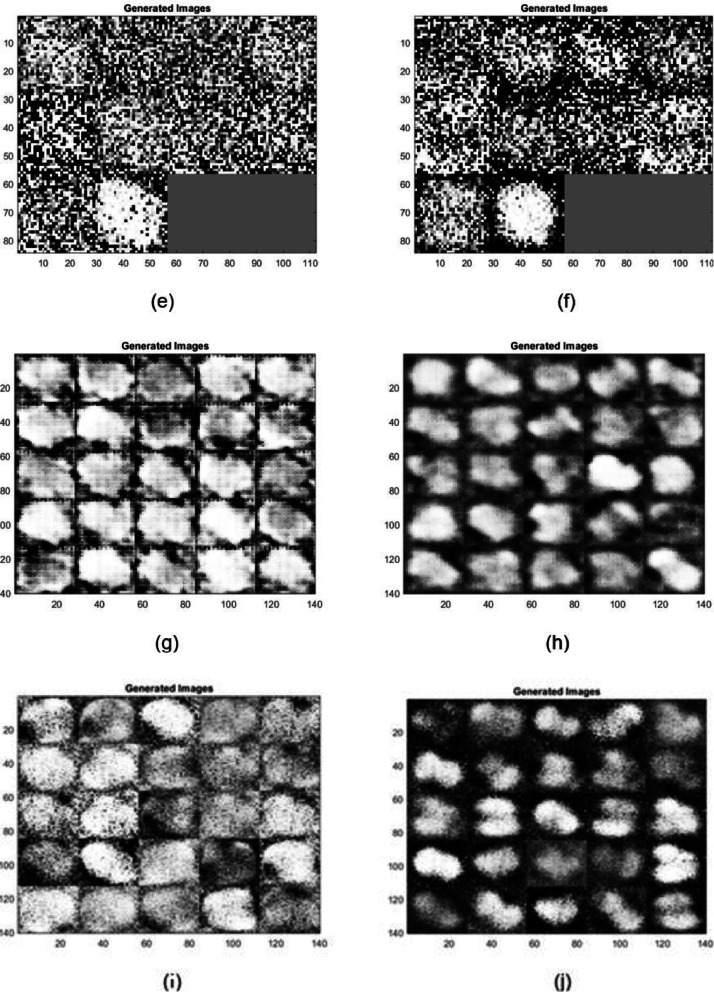

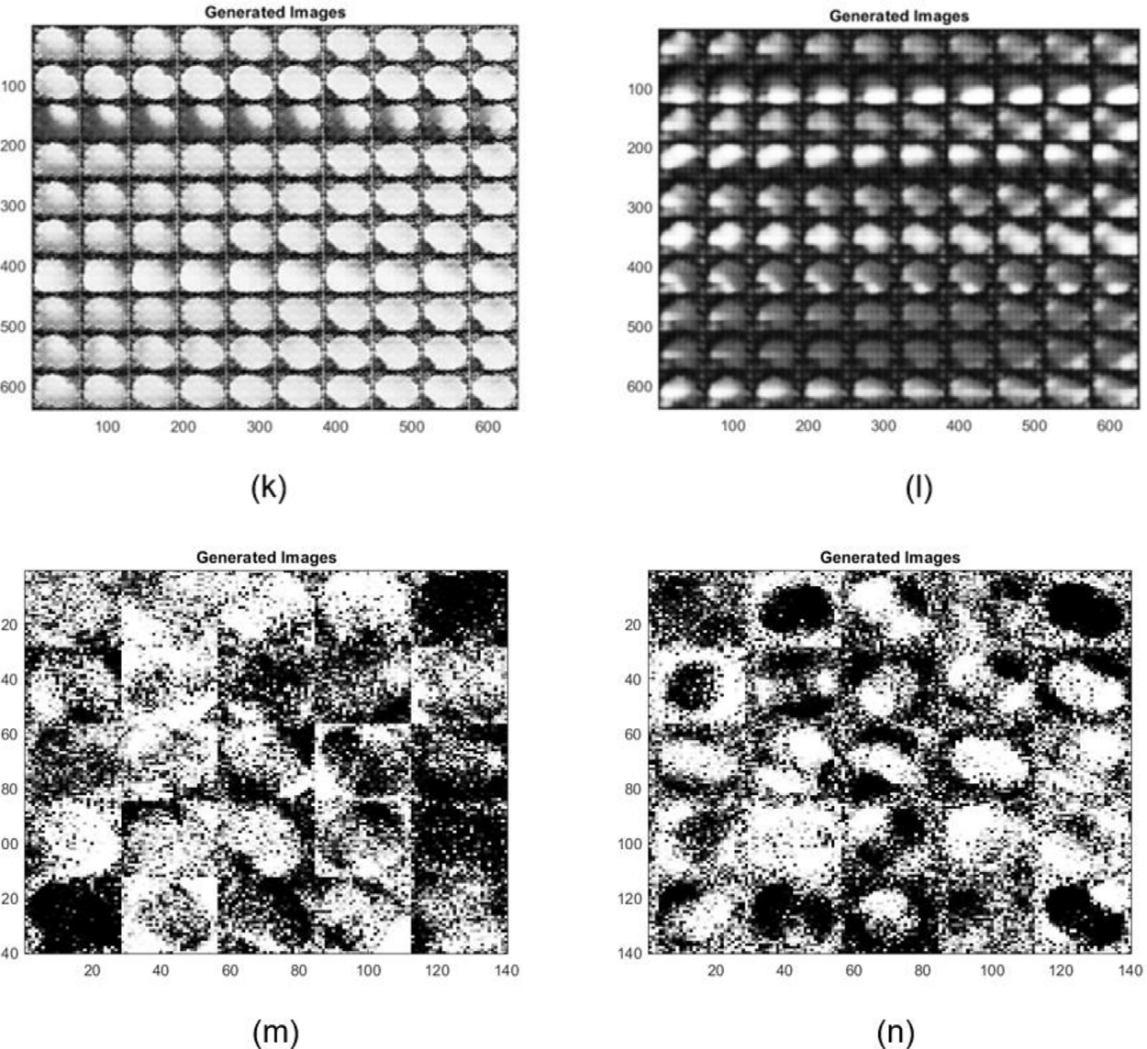
*Raphanus raphanistrum* instances generated by different GAN architectures: (a) AAE - RGB (b) AAE–IR (c) CGAN–RGB (d) CGAN–IR (e) CGAN–RGB (f) CGAN–IR (g) DCGAN–RGB (h) DCGAN–IR (i) InfoGAN - RGB (j) InfoGAN–IR (k) WGAN–RGB (l) WGAN–IR (m) LSGAN–RGB (n) LSGAN–IR.

The generated images demonstrate varying quality and detail fidelity across different GAN architectures, which can be attributed to the complexity of the datasets and the differences in architectural enhancements (Fig. 7). The Modified WGAN-GP displayed higher fidelity when generating both RGB and infrared (IR) images. The generated RGB images maintained clearer structural details, such as well-defined boundaries and texture, which indicates the capability of the modified WGAN-GP to effectively capture and replicate the original dataset's essential characteristics. In contrast, the IR images generated more variability, with noticeable fluctuations in quality across different GAN models, suggesting increased sensitivity and challenges when dealing with complex spectral information.

The comparative performance of various GAN architectures, observed through SSIM trends across epochs, highlights that the Modified WGAN-GP and Auxiliary Classifier GAN (ACGAN) generally fared well compared to other GAN models for both RGB and IR data (Fig. 5, Fig. 6). The IR dataset, however, was observed to be more challenging for reconstruction, resulting in lower SSIM values for most models, which points to the inherent difficulty of preserving structural similarity in infrared imagery. The ACGAN achieved the highest SSIM values for both RGB and IR, suggesting robust capability in maintaining image quality (Fig. 7). Other models like CGAN and LSGAN struggled with both modalities, likely due to limitations in capturing fine details, as reflected in consistently low SSIM scores.

Overall, the observations indicate that architectural enhancements, such as gradient penalty and auxiliary tasks, can play a crucial role in improving the quality of generated images, especially for datasets with high spectral complexity like IR. Future improvements can be based on further refining these models, particularly by addressing the challenges associated with IR data generation, enhancing stability during training, and possibly integrating newer techniques such as attention mechanisms to improve consistency and quality across modalities.

### Limitations

The architectural modifications demonstrated in this study are limited to just two spectral modalities of *Raphanus raphanistrum* datasets. With minor adjustments, this approach can be applied to other agricultural weeds. These advancements will ultimately support better crop management, reduce environmental impacts, and improve decision-making in precision agriculture and related fields.

The success of modified WGAN-GP is largely attributed to its architectural customizations, such as the incorporation of gradient penalties, convolutional layers, and batch normalization. These modifications contribute to enhanced training stability and improved image quality, particularly for RGB datasets. Similarly, ACGAN leverages auxiliary tasks to bolster feature reconstruction and adaptability across diverse datasets, leading to promising and consistent performance. Despite these advancements, the generation of high-quality IR images continues to pose challenges. This variability in IR image quality underscores the need for further research focused on preserving structural integrity in this modality.

## Discussion

GANs have been used to create realistic synthetic images, to address the lack of labelled data in crop/weed segmentation tasks. This enhances the training of segmentation networks, improving their ability to distinguish between crops and weeds [38,39]. AI systems can accurately identify and target weeds, reducing herbicide usage and minimizing environmental impact. For instance, AI-powered systems have demonstrated over 90 % accuracy in eradicating weeds, offering sustainable alternatives to traditional chemical methods [40]. Despite these advancements, challenges such as the need for extensive labelled datasets, environmental variability, and the adaptability of AI models to diverse agricultural settings persist. Ongoing research aims to address these issues by developing more generalized models and integrating advanced sensing technologies [14]. In summary, the integration of GANs into precision agriculture holds substantial promise for enhancing weed management practices. By generating synthetic data for training robust models, GANs can contribute to more efficient, accurate, and sustainable agricultural practices.

Collectively, these studies underscore the need for more comprehensive GAN frameworks that can handle spectral variation, ensure contextual coherence in synthetic scenes, and enhance the representation of weeds. Addressing these challenges is critical for the next generation of synthetic data pipelines in precision agriculture. Computer vision experts can explore enhancements to this model to address challenges associated with IR data generation, improve training stability, and integrate newer techniques, such as attention mechanisms, to enhance consistency and quality across modalities.

## Conclusion

This study introduced a modified WGAN-GP architecture that advances data augmentation techniques to address the need for diverse, representative datasets in training agricultural AI systems. By ensuring cross-modal coherence (RGB and infrared/IR) and adaptability to real-world environmental heterogeneity, the architecture enhanced the generation of synthetic images which closely resembled complex field conditions. Beyond demonstrating potential to the existing GANs, our work redefined synthetic data quality in contextual coherence and not just visual fidelity. This is critical for training robust field-deployable models. Practically, this reduces dependency on scarce annotated field data. Theoretically, it motivates the AI community to prioritize domain-specific architectures over generic models, particularly in multi-sensor environments. Future research should consider hybrid models, such as combining GANs with Variational Autoencoders or diffusion models, to further improve fidelity and versatility. The limitations of these customizations highlight the need for spectra-specific attention mechanisms, advanced loss functions, and spectral normalization to address the unique complexities of IR data. The overall experiment emphasized the importance of the proposed model's generalizability and experimentation over *Raphanus raphanistrum* samples provided a controlled framework for evaluation.

## Ethics statements

This research did not involve human subjects, animal experiments, or data collected from social media platforms. Therefore, no specific ethical approvals or informed consent were required for this study.

## CRediT author statement

**Shubham Rana:** Conceptualization, Methodology, Validation, Data curation, Writing-Original draft preparation, Editing. **Matteo Gatti:** Methodology, Editing, Validation, Supervision, Reviewing.

## Declaration of competing interest

The authors declare that they have no known competing financial interests or personal relationships that could have appeared to influence the work reported in this paper.

## Data Availability

https://doi.org/10.5281/zenodo.10567783.
